# Jean-François Borel (1933–2025)

**DOI:** 10.3389/ti.2025.15303

**Published:** 2025-08-20

**Authors:** Pierre-Alain Clavien

**Affiliations:** Department of Surgery and Transplantation, University of Zurich and Swiss Medical Network, Zurich, Switzerland

**Keywords:** organ transplantation, cyclosporin A, organ transplant, rejection, immunity

Transplant International mourns the profound loss of Jean-François Borel, who significantly advanced the field of solid organ transplantation through his breakthrough work on Cyclosporin.

Jean-François Borel, a Swiss microbiologist and immunologist, was born on 4 July 1933, in Belgium. After moving to his home country Switzerland during the second world war he studied at the Swiss Federal Institute of Technology in Zurich, where he earned his Ph.D. in immunological genetics in 1964. JF Borel’s curiosity and passion for science and art drove him to pursue a career that would change the face of medicine.

JF Borel’s most notable contribution was in the discovery of cyclosporin, a drug that has transformed the field of organ transplantation. His research demonstrated the drug’s ability to selectively suppress T-cells, paving the way for its use in humans. Cyclosporin’s impact on transplantation medicine has been profound, enabling hundreds of thousands of people to receive life-saving organ transplants.

Throughout his illustrious career, JF Borel received numerous honors for his work, including the Gairdner Foundation International Award, the Paul Ehrlich and Ludwig Darmstaedter Prize, as well as the Cloëtta Prize. He was also awarded an honorary doctorate from the University of Basel.

Beyond his scientific achievements, JF Borel was a man of diverse passions and talents. One of his greatest loves was painting (Figure 1). JF Borel’s artwork reflects his keen eye for detail and his ability to capture the beauty in the world around him. His paintings often incorporated elements of nature, showcasing his deep appreciation for the natural world. Through his art, JF Borel expressed himself in a different way, exploring new ideas and perspectives that complemented his scientific pursuits. His love of painting brought him joy and fulfillment, and his artwork remains a testament to his creativity and talent.

**FIGURE 1:**
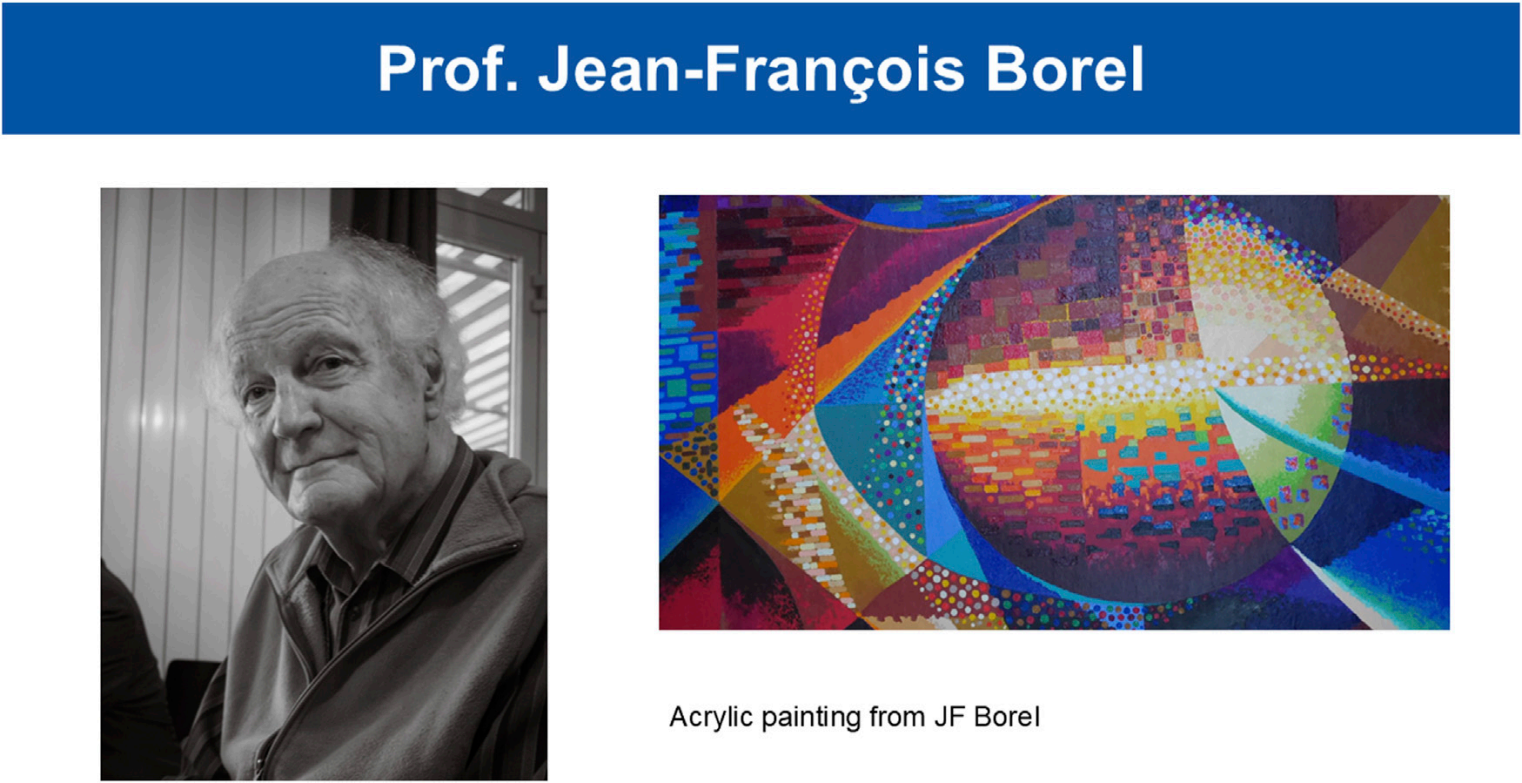
Prof. Jean-François Borel and Acrylic painting from JF Borel, courtesy of Patrick Borel.

Since 2000 we hold a yearly special lecture in honor of JF Borel at the University Hospital in Zurich, where leaders and innovators in transplantation medicine give a special lecture. While Jean François Borel withdrew from the scientific public life, he regularly attended his namesake lecture in Zurich, and spent his time enjoying art, literature and of course painting.

JF Borel’s life was a testament to the idea that we are all multifaceted individuals, with many passions and interests that shape who we are. His love of science and art reminds us that these two seemingly disparate fields are, in fact, interconnected, and that together, they can lead to a richer, more fulfilling life.

As the scientific community and many patients around the globe mourn his passing, we take comfort in the knowledge that JF Borel’s legacy will live on. His work will continue to inspire future generations of researchers, and his artwork will bring joy and inspiration to those who see it. His passing leaves a void, but his impact will endure, a reminder of the difference one person can make in the world.

Pierre-A. Clavien.

## Data Availability

The original contributions presented in the study are included in the article/supplementary material, further inquiries can be directed to the corresponding author.

